# The Effect of Inorganic Filler Content on the Properties of BPA-Free Bulk-Fill Dental Resin Composites

**DOI:** 10.3390/ma17205040

**Published:** 2024-10-15

**Authors:** Huilin Deng, Fang Liu, Jingwei He

**Affiliations:** School of Materials Science and Engineering, South China University of Technology, Guangzhou 510641, China; 202320118059@mail.scut.edu.cn (H.D.); mcfliu@126.com (F.L.)

**Keywords:** bulk-fill resin composites, inorganic filler content, physicochemical properties

## Abstract

This study aimed to enhance the performance of dental resin composites (DRCs) by increasing the content of inorganic fillers while addressing potential health risks associated with Bisphenol A (BPA). To achieve this, the BPA-based resin monomer Bis-GMA was replaced with BPA-free Bis-EFMA. The study then explored the impact of varying inorganic filler contents on the physiochemical properties of Bis-EFMA-based bulk-fill dental resin composites (BF-DRCs). Four distinct Bis-EFMA-based BF-DRCs were formulated, each with different inorganic filler contents ranging from 70 wt% to 76 wt%. The study tested the depth of cure (DOC), double-bond conversion (DC), water sorption (WS), solubility (SL), and cytotoxicity of the system. It notably investigated the effects of increasing filler content on mechanical properties through flexural strength (FS), flexural modulus (FM), Vickers microhardness (VHN), and wear resistance, as well as the impact on polymerization shrinkage, including volumetric shrinkage (VS) and shrinkage stress (SS). To assess the commercial application potential of Bis-EFMA-based BF-DRC, the research used the commercially available BF-DRC Filtek Bulk-Fill Posterior (FBF) as a control. The results indicated that a higher filler content did not affect the DOC of Bis-EFMA-based BF-DRCs. Inorganic fillers at higher concentrations significantly enhanced overall mechanical properties while significantly reducing volumetric shrinkage (VS; *p* < 0.05). When the concentration of inorganic fillers in the resin system reached 76 wt%, most of the performance of the Bis-EFMA-based BF-DRC surpassed that of the commercial control FBF, except for FS, FM, and SS. These findings highlight the potential of Bis-EFMA-based BF-DRC as a long-term restorative material for dental applications.

## 1. Introduction

Resin-based materials have gained significant traction in various dental applications. They are most commonly used in dental prosthetics, where dental resin composites (DRCs) that match tooth color adhere to dental tissue using adhesives. This advancement has led to DRCs replacing amalgam fillings as the preferred solution for repairing wear, trauma, or caries [1]. DRCs consist of two main components, including resin matrix (containing monomer, photopolymerization initiators, promoter, inhibitor, and colorant) and inorganic filler particles, which provide most of the DRCs’ mechanical properties and the desired optical and radiopaque properties required [2]. Common monomers in DRCs consist mainly of a mixture of monomers, including Bisphenol A glycidyl methacrylate (Bis-GMA), urethane dimethacrylate (UDMA), and triethyleneglycol dimethacrylate (TEGDMA).

Despite their successful application in dental practices for several decades, concerns regarding the safety and biocompatibility of dental resin composites (DRCs) persist. These concerns are primarily attributed to the release of incompletely polymerized and unpolymerized compounds, including residual monomers, which have the potential to diffuse through dentinal tubules, be absorbed into the pulp, or be released into the oral cavity and subsequently ingested [3]. Furthermore, the release of Bisphenol A (BPA), an endocrine disruptor, and the associated health risks have garnered significant attention [4,5,6]. Numerous studies have indicated that BPA, a degradation product present in most dental resin composites, can lead to severe health issues, such as fetal growth restriction [7], anxiety and depression [8], obesity [9], and adverse immune effects [10]. Bis-GMA, a BPA derivative, is widely used in DRCs due to its favorable mechanical properties post-polymerization, low volumetric shrinkage, low volatility, and high refractive index [11]. However, removing this monomer from resin formulations and reducing resin content may help mitigate the health risks associated with BPA [12]. Although UDMA, a non-BPA derivative monomer, can partially or fully substitute Bis-GMA, UDMA-based dental resin composite exhibit higher volumetric shrinkage compared to those containing Bis-GMA [13]. Therefore, further research is necessary to explore alternative monomers to Bis-GMA that can provide similar or improved properties without the associated health risks [14,15,16,17].

He and his colleagues developed a BPA-free high-refractive-index resin monomer, called Bis-EFMA [18]. This innovative monomer boasts a molecular weight and rigid phenyl structure that contribute to enhanced mechanical properties. Its high refractive index aligns well with fillers, improving light transmission in DRCs. Bis-EFMA serves not only as an excellent alternative to Bis-GMA but also as a resin monomer for producing bulk-fill dental resin composites (BF-DRCs), which are designed for placement in increments of 4 or 5 mm, enabling efficient one-step curing [19]. BF-DRCs can overcome time-consuming incremental placement techniques, offering low polymerization stress and outstanding physical properties, excelling in wear resistance, functionality, and aesthetics [20]. Numerous studies have validated the effective performance of the Bis-EFMA resin monomer. Hatipoğlu et al. investigated the elution, microhardness, and roughness of Bis-EFMA-based DRCs, concluding that Bis-EFMA serves as a viable alternative to Bis-GMA in commercial DRCs due to its superior color stability [21], reduced monomer elution, and increased microhardness [22]. Zhang et al. formulated resin systems utilizing Bis-EFMA and TEGDMA in varying proportions, which demonstrated superior refractive indices over Bis-GMA-based systems, alongside improved flexural modulus and minimized volumetric shrinkage and shrinkage stress, enabling functionality as BF-DRCs [23]. Ma et al. examined various properties of DRCs incorporating Bis-EFMA by mixing five distinct ratios of UDMA, revealing that the depth of cure of these composites meets the requirements for BF-DRCs with suitable resin formulations. Furthermore, specific ratios of Bis-EFMA/UDMA-based DRCs offer benefits, such as enhanced flexural modulus and flow, decreased polymerization shrinkage, exceptional biocompatibility, and favorable adhesive properties, compared to Bis-GMA-based DRCs [24]. In clinical assessments, the incorporation of Bis-EFMA demonstrated remarkable biocompatibility and color stability. It provided edge-sealing performance comparable to the clinically utilized BF-DRCs, along with commendable mechanical strength and wear resistance [25]. With these impressive attributes, Bis-EFMA is poised for application in commercial DRCs, particularly those designated for use as BF-DRCs.

Safe and efficient resin composites are among the research objectives in dental materials. Building upon the foundational work conducted on Bis-EFMA, this study set its sights on propelling the commercial application of Bis-EFMA-based DRCs by augmenting their mechanical properties and biocompatibility. The study delves into the ramifications of augmenting the inorganic filler content within Bis-EFMA-based DRCs. This extensive research explores crucial parameters for evaluating the performance of resin composite. It examines aspects such as the depth of cure (DOC), double-bond conversion (DC), water sorption (WS), solubility (SL), and cytotoxicity. The study primarily focuses on how augmenting the content of inorganic fillers influences the mechanical properties of BF-DRCs. It includes features such as flexural strength (FS), flexural modulus (FM), Vickers microhardness (VHN), and wear resistance, along with the effects on polymerization shrinkage. To test the validity of our assumptions, we formulated three null hypotheses: (1) Elevating the inorganic filler content would alter the bulk filling performance of Bis-EFMA-based DRCs, (2) augmenting the inorganic filler content would not exert a significant influence on the mechanical properties of Bis-EFMA-based DRCs, and (3) enhancing the inorganic filler content would not notably affect the polymerization shrinkage of Bis-EFMA-based DRCs. Through rigorous experimentation and analysis, this study aims to uncover the optimal balance of inorganic filler content that would propel Bis-EFMA-based BF-DRCs to the forefront of dental material innovation, combining good operability, exceptional mechanical strength, and minimal polymerization shrinkage.

## 2. Materials and Methods

### 2.1. Materials

**Figure 1:**
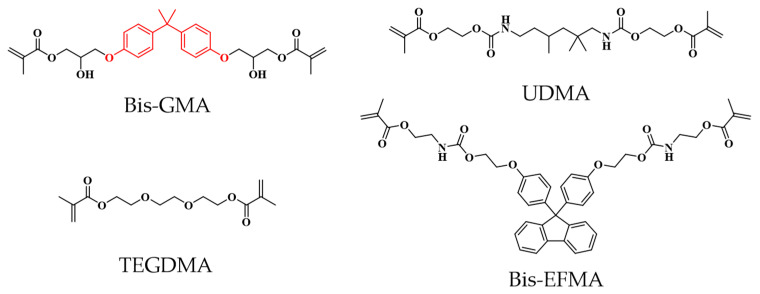
The structures of the resin monomer [13,18]. In the structural formula of Bis-GMA, the red-highlighted section represents the core structure of Bisphenol A. The chemical formulas were drawn using ChemDraw 18.0.

The 9,9-Bis[4-((2-(2-methacryloyloxy)ethyl-carbamate)ethoxy)phenyl] fluorene (Bis-EFMA) was prepared by the method provided in the literature [18] (Figure 1). Triethyleneglycol dimethacrylate (TEGDMA) and urethane dimethacrylate (UDMA) were sourced from the Tokyo Chemical Industry, Tokyo, Japan. Camphor quinone (CQ) was acquired from J&K Scientific, Beijing, China. N, N′-dimethylaminoethyl methacrylate (DMAEMA) was procured from J&K Scientific, Beijing, China. Silanated inorganic glass fillers (SCHOTT^®^ UltraFine, GM 27884, with 6% silane, a particle size of 0.7 μm, and a refractive index of 1.53) were obtained from SCHOTT AG in Mainz, Germany. As a reference material, 3 M™ Filtek™ Bulk-Fill Posterior (FBF) was purchased from 3 M Espe in St. Paul, MN, USA. The curing light used was the CL 2500, which operates in a wavelength range of 400–520 nm and at an intensity of approximately 550 mW cm⁻^2^, manufactured by 3 M Co., MN, USA.

### 2.2. Preparation of Dental Resin Matrix and Composites

**Figure 2:**
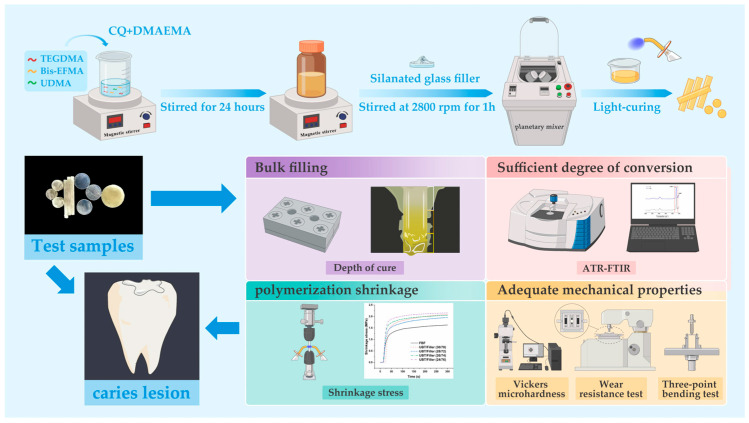
Schematic diagram of the preparation and testing of DRCs.

The dental resin systems without fillers were formulated based on the proportions detailed in Table 1. Each component was carefully weighed and thoroughly mixed using a magnetic stirrer. The resin matrix composition was determined with reference to prior research [23,24], consisting of UDMA, Bis-EFMA, and TEGDMA in weight percentages of 36 wt%, 24 wt%, and 40 wt%, respectively. To prepare the DRCs, silanated inorganic glass fillers were blended with the resin matrix using a high-speed mixer (ZHI DI TECH, Shenzhen, China) at 2800 rpm until a homogeneous mixture was achieved (Figure 2). For comparison, FBF was selected as the commercial control material, which features a matrix composed of Bis-GMA, UDMA, Bisphenol A ethoxylate dimethacrylate (Bis-EMA), TEGDMA, and EDMAB (RI: 1.52), and filler content of silica, zirconia, and YbF_3_, at a weight/volume ratio of 64.5/42.5 wt%/vol%.

### 2.3. The Mixing Degree

After being subjected to light curing, the DRCs were deliberately fractured in liquid nitrogen to reveal their internal structure. Following the application of a gold coating, the extent of mixing between the resin matrix and inorganic fillers was meticulously examined on the newly exposed cross-section using a high-resolution scanning electron microscope (SEM; Merlin, Zeiss, Germany) operating at a voltage of 5 kV. This allowed for a detailed analysis of the composite’s microstructure.

### 2.4. Double-Bond Conversion (DC)

Double-bond conversion of the BF-DRCs modified with different filler contents was evaluated by attenuated total reflection Fourier transform infrared spectroscopy (ATR-FTIR; Thermo Scientific Nicolet iS50, Thermo Fisher Scientific Inc., Waltham, MA, USA) using an attenuated total reflectance device with a diamond crystal. The uncured resin composite sample was filled into a cylindrical silicon mold with thicknesses of 2 mm, 3 mm, 4 mm, and 5 mm, and a diameter of 5 mm. Infrared light emitted from a source scanned the samples from the bottom to the top within a range of 4000 cm^−1^ to 400 cm^−1^, with 4 scans measured per sample (resolution: 4 cm^−1^). Based on the normalized peak area of the characteristic absorbance peaks associated with the polymerizable groups on the resin monomers, the DC of the ATR-FTIR spectra for samples with different thicknesses was evaluated using Equation (1):

(1) $$\mathrm{DC}\left(\%\right)=\left[1-\frac{{\left(A_{C=C}/A_{C=O}\right)}_{t}}{{\left(A_{C=C}/A_{C=O}\right)}_{0}}\right]\times 100$$

**Figure 3:**
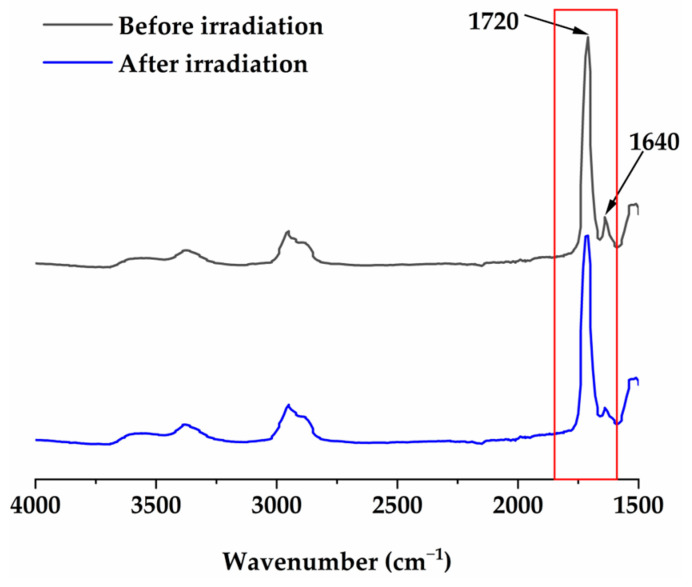
ATR-FTIR spectra of DRCs before and after irradiation at 1636 and 1720 cm^−1^.

In the absorption spectrum, *A_C=C_* at 1640 cm^−1^ and *A_C=O_* at 1720 cm^−1^ correspond to the peak areas of the characteristic and standard peaks (as shown in Figure 3). The subscripts t and 0 correspond to irradiation times of 40 s and 0 s (uncured state), respectively.

### 2.5. Depth of Cure (DOC)

DOC was measured following the method noted in ISO 4049:2019(E) [26]. The uncured samples were filled into a cylindrical stainless-steel mold, which had a diameter of 4 mm and a height of 15 mm. After 20 s and 40 s of light exposure, the samples were removed, and their uncured bottoms were scraped off with a plastic scraper. The height of the cured portion of the resin was measured using a spiral micrometer, and half of the measurement was taken as DOC.

### 2.6. Vickers Microhardness (VHN)

Cylindrical stainless-steel molds, with an inner diameter of 7 mm and heights ranging incrementally from 2 mm to 5 mm, were utilized to prepare the samples for VHN testing. All samples were irradiated from above for 40 s and subsequently stored in darkness at room temperature for 24 h before testing.

A Vickers microhardness tester (model HL-101, manufactured by Dongguan Magnanimity Instrument Equipment Co., Ltd. in Dongguan, China) was employed to measure the VHN of the DRC samples. To ensure accurate results, three samples from each group were tested, and for each sample, both the top and bottom surfaces were examined. Three random positions were selected and tested around the center of the cured sample at each surface level to account for potential variations in VHN and the crosslinking density across the specimen’s surface. A load of 500 gf (with a dwell time of 5 s) was applied at each position, and the mean VHN value per surface level was determined from the three readings obtained. The ratio of the initial VHN values at the bottom and top surfaces was calculated to assess the depth-dependent effectiveness of the curing process [27].

### 2.7. Volumetric Shrinkage (VS)

Volumetric polymerization shrinkage was assessed by measuring density changes using Archimedes’ principle. All the samples were weighed several times in two different environments of recognized density (conventional air and distilled water). Uncured samples were contained in 5 mL glass cups to facilitate density measurement. Equation (2) determined the density of the pre-photopolymerized sample (*ρ_ps_*), the density of the empty cup (*ρ_ec_*), and the overall density of the cup containing the uncured sample (*ρ_cc_*):

(2) $$\rho =\frac{m_{a}}{m_{w}-m_{a}}\rho_{T}$$

where *ρ*, *m_w_*, *m_a_*, and *ρ_T_* are the density of the sample, weight of the sample in water, weight of the sample in air, and density of water at the measured temperature. The density of the uncured sample (*ρ_us_*) was determined using Equation (3):

(3) $$\rho_{us}=\frac{m_{cca}-m_{eca}}{\frac{m_{cca}}{\rho_{cc}}-\frac{m_{eca}}{\rho_{ec}}}$$

where *ρ_uc_*, *m_cca_*, *m_eca_*, *ρ_cc_*, and *ρ_ec_* are the density of the uncured sample, the weight of the cup containing the uncured sample in air, the weight of the empty cup in air, the density of the cup containing the uncured sample, and the density of the empty cup.

Volume shrinkage (VS) can be calculated according to Equation (4), where *ρ_us_* and *ρ_ps_* are the sample density before and 40 s after photopolymerization:

(4) $$VS\left(\%\right)=\left(1-\frac{\rho_{us}}{\rho_{ps}}\right)100$$

### 2.8. Shrinkage Stress (SS)

To measure the polymerization shrinkage stress (SS) of DRCs, two cylindrical glass fiber rods, each with a diameter of 6 mm and a length of 4 cm, were securely fastened in the universal testing machine (Z010, Zwick-Roell GmbH & Co. KG, Ulm, Germany) using a gripping device. Both ends of the rods were ensured to be uniformly flat and lightly coated with a layer of adhesive. The uncured resin composite was then carefully filled into the 1 mm gap between the rods, and any excess resin that squeezed out was scraped away. Two curing lamps, positioned 5 mm away from the resin composite on both sides, were used to irradiate the sample vertically for 20 s, simultaneously. At room temperature, the polymerization shrinkage force of the resin composite was measured and recorded for 300 s, generating a curve that depicted the variation in the force over time, along with the maximum polymerization shrinkage force value. The method for calculating the polymerization shrinkage stress is outlined in Equation (5):

(5) $$SS\left(MPa\right)=\frac{4F_{max}}{\pi d^{2}}$$

where *F*_max_ (N) signifies the peak load encountered during the testing phase, whereas *d* (mm) indicates the diameter of the glass fiber rod. For each DRC, the above procedure was repeated five times.

### 2.9. Flexural Strength (FS) and Flexural Modulus (FM)

FS was assessed using the three-point bending test. Rectangular samples, measuring 2 mm × 2 mm × 25 mm, were utilized for this analysis, adhering to the ISO 4049:2019(E) [26] standard. Each group comprised twenty samples. Ten samples remained dry before testing, while the remaining samples were immersed in 35 mL of distilled water at 37 ± 1 °C until evaluation. A universal testing machine (AGS-10KN, Shimadzu Corporation, Kyoto, Japan) conducted the measurements at the speed of 1 mm/min. During the process, the FM of elasticity was also evaluated. According to ISO 4049 [26], DRCs were required to exhibit an FS of 80 MPa to comply with the criteria for restorative materials.

### 2.10. Water Sorption (WS) and Solubility (SL)

To evaluate WS and SL, the samples were prepared using silicone molds (15 mm diameter and 1 mm width). We applied tested materials in a single layer and irradiated them in nine non-overlapping zones, adhering to ISO 4049:2019(E) [26]. Eight samples were prepared for each DRC, stored in a desiccator to eliminate moisture, and their weight was recorded (FA1104J, Shunyuhengping Scientific Instrument Co., Ltd., Shanghai, China) at regular intervals until we achieved a constant mass (m_1_). Dried samples were weighed (*m*_1_) on a digital scale, which offered a minimum precision of 0.1 mg. After immersing them in 30 mL of distilled water at (37 ± 1) °C for 7 days, we weighed the samples’ mass (*m*_2_). Next, we dried the samples at 80 °C until their mass stabilized (*m*_3_). WS and SL were calculated according to Equations (6) and (7):

(6) $$WS\left(\mu g/mm^{3}\right)=\frac{m_{2}-m_{1}}{V}$$

(7) $$SL\left(\mu g/mm^{3}\right)=\frac{m_{1}-m_{3}}{V}$$

where *m*_1_ represents the initial mass of the sample, *m*_2_ denotes the mass of the sample after water sorption, *m*_3_ indicates the mass of the dried sample post-soaking, and *V* signifies the initial volume of the dried sample.

### 2.11. Wear Resistance

The MFT-4000 multifunctional material surface performance tester (MFT-4000, Lanzhou Huahui Instrument Technology Co., Lanzhou, China) facilitated reciprocating friction and wear assessments of DRC sample surfaces in ambient air conditions. The sample dimensions were 15 mm in width and 4 mm in height, polished on a water mill with 3000 grit silicon carbide sandpaper at a speed of 120 rpm for 30 min. This process ensured that both the top and bottom surfaces were parallel, smooth, and free of visible scratches. Subsequently, these samples were mounted on a high-speed reciprocating wear resistance testing machine, exposing the sample surface to reciprocating friction. ZrO_2_ ceramic balls, with a 4 mm diameter and hardness akin to human teeth, were utilized as the friction pair. Modeling the normal chewing process in the oral cavity—with saliva serving as a lubricant—the chewing force was set between 3 and 36 N [28]. The load was established at 25 N, with a reciprocating speed of 200 mm/min and a length of 5 mm, all while maintaining ambient air conditions as the friction medium. Each sample underwent testing for 60 min. After the experiment, an optical profilometer (RTEC UP Dual Model, Rudolph Technologies, Inc., Wilmington, MA, USA) assessed the surface morphology, depth, and volume of grooves for each sample group under a 20× microscope.

### 2.12. Measurement of Cytotoxicity

We prepared the cell culture medium following ISO 10993-5 standards [29]. We incorporated 10% fetal bovine serum and 1% penicillin/streptomycin (100 units per milliliter) into the MEM basic medium (Gibco, Thermo Fisher Scientific, Waltham, MA, USA). Next, we fabricated cylindrical samples measuring 10 mm in diameter and 1 mm in height for each DRC. We then sterilized all samples by exposing them to UV light on each side for 1 h, then immersed them in the designated cell culture medium with a specific surface area to medium volume ratio of 1.25 cm^2^/mL. Samples were stored in a 37 °C incubator for 24 h, filtered through a 0.22 μm sterile filter, and subsequently transferred to −4 °C for preservation. We cultivated L-929 mouse fibroblast cells (ICell-M026, Guangzhou Yanshi Technology Co., Ltd., Guangzhou, China) in a cell culture incubator at 37 °C under 5% CO_2_ humidified air. A trypsin substitute (Tryp^TM^ Express, Thermo Fisher Scientific, USA) was used to dissociate the cells; then, we placed them on a 96-well plate at a density of 6000 cells per well, using a medium volume of 100 μL per well. After 24 h of incubation, the experimental group’s medium was replaced with the sample extract, applying three concentration gradients of the extract: 100%, 50%, and 25% (diluted with cell culture medium), while the control group’s medium was replaced with fresh cell culture medium as a negative control. This was followed by a 24 h incubation in the 37 °C cell culture incubator before evaluating cell viability using the Cell Counting Kit-8 (CCK-8, Dojindo Molecular Technology, Tokyo, Japan). After administering 10 μL of CCK-8 to each well, the samples were incubated for 2 h, then the absorbance was measured at 450 nm with a microplate reader (BioTek EPOCH_2_, BioTek Instruments, Inc., Winooski, VT, USA). We calculated cell viability (CV) using Equation (8):

(8) $$CV\left(\%\right)=\frac{OD_{e}-OD_{b}}{OD_{c}-OD_{b}}\times 100$$

where *OD_e_* indicates the absorbance of the well containing treated cells exposed to the extract and CCK-8. *OD_b_* represents the absorbance of the well containing only medium and CCK-8. Lastly, *OD_c_* shows the absorbance of the well with untreated cells incubated in the medium with CCK-8.

### 2.13. Statistical Analysis

The statistical analysis of all data was conducted using SPSS 26.0 (IBM SPSS Statistics 26.0.0.0, Chicago, IL, USA). For inter-group data analysis, one-way analysis of variance (ANOVA) and Tukey’s post hoc comparison test were employed to assess statistical differences between groups, with *p* < 0.05 indicating significant differences. When the number of samples between groups was less than three, an independent samples T-test was utilized, and after verifying homogeneity of variance, *p* < 0.05 also indicated significant differences.

## 3. Results

**Figure 4:**
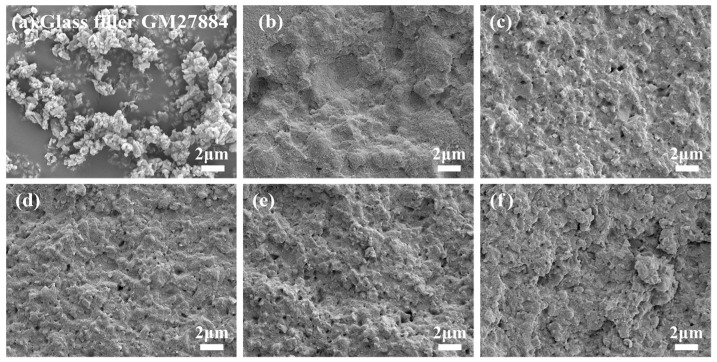
SEM images depict the micro-filler particles utilized as additional fillers for BF-DRCs: (**a**) SCHOTT^®^ UltraFine silanated inorganic glass fillers, and the section morphology of DRCs: (**b**) DRCs of FBF, (**c**) DRCs of UBT/Filler (30/70), (**d**) DRCs of UBT/Filler (28/72), (**e**) DRCs of UBT/Filler (26/74), and (**f**) DRCs of UBT/Filler (24/76).

This electron microscope analysis revealed that all inorganic filler particles and resin matrix added to the BF-DRCs were successfully combined in different DRCs, as shown in Figure 4.

The results for DOC, WS, and SL are shown in Table 2. We can notice how UBT/Filler (30/70) and UBT/Filler (24/76) presented a deeper depth of cure (40 s) compared to FBF, with an average DOC of 5.589 mm in a range between 5.389 and 5.789 mm for UBT/Filler (30/70) and a mean value of 5.660 mm in a range between 5.460 and 5.860 mm for UBT/Filler (24/76). There was no significant change in the DOC of the experimental groups with increasing filler content when the curing time was extended from 20 s to 40 s (*p* > 0.05). The WS of the experimental groups presented a significant decrease (*p* < 0.05) with increasing filler content, and there was no significant difference in SL (*p* > 0.05). The commercial control FBF had no significant difference in DOC (20 s) over the experimental group (*p* > 0.05), while the FBF had higher WS and SL (*p* < 0.05).

Mean values and standard deviations of VHN registered with the samples (thicknesses of 2, 3, 4, and 5 mm) for the top and bottom surfaces are listed in Table 3. We considered the five bulk-fill dental resin composites (FBF, UBT/Filler (30/70), UBT/Filler (28/72), UBT/Filler (26/74), and UBT/Filler (24/76)) and compared their hardness ratios, in accordance with the external (top) versus the internal side (bottom) of the samples, as shown in Table 3. Despite decreased values observed between the top and bottom surfaces with increased sample thicknesses, the hardness ratio exceeded 0.80 across all groups. Statistical analysis of variance (ANOVA) showed that all experimental groups had a significant VHN increase (*p* < 0.05) with the increasing filler content, under the same thickness of sample conditions. With the proportion of 24/76 (UBT/Filler), it showed much higher (*p* < 0.05) VHN than FBF regardless of thicknesses.

Mean values and standard deviations of DC results of the samples at different thicknesses (thicknesses of 2, 3, 4, and 5 mm) are listed in Table 4. The DC of DRCs in the experimental groups with different filler content was not significantly different at each sample thickness (*p* > 0.05). When the thickness of all DRC samples was increased to 5 mm, the DC decreased significantly, except for UBT/Filler (30/70; *p* < 0.05).

**Figure 5:**
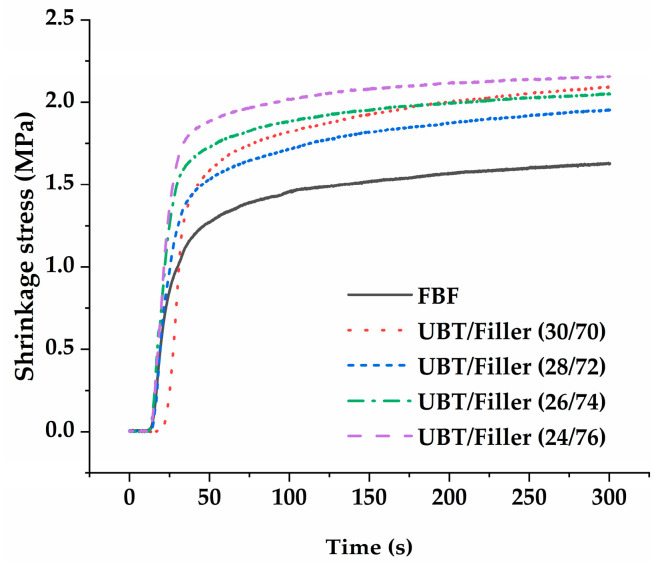
Shrinkage stress versus time curves of DRCs.

The tested materials’ mean SS (±SD), expressed in MPa, as well as mean VS (±SD) are shown in Table 5. Representative SS versus time curves are shown in Figure 5. The statistical analysis of variance for SS evaluation showed that FBF presented significantly lower stress during irradiation than the other tested materials (*p* < 0.05). For the experimental groups, no significant difference in the SS values of the DRCs was observed with the increasing filler content (*p* > 0.05). The VS of all experimental groups decreased with the increase in filler in the resin system (*p* < 0.05). UBT/Filler (26/74) and UBT/Filler (24/76) showed much lower (*p* < 0.05) VS than FBF. Figure 5 illustrates that the SS of all DRCs initially increased rapidly and then gradually leveled off. However, during the first 20 s, as the inorganic filler content in the system increased, the initial increase rate of DRC curing accelerated significantly, and except for UBT/Filler (30/70), the final SS showed a notable upward trend.

**Figure 6:**
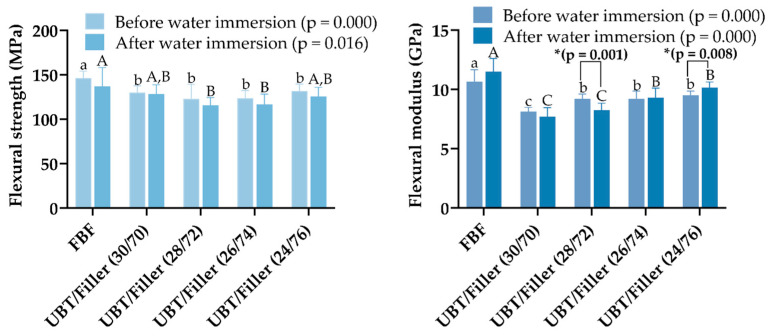
Flexural strength and modulus before and after water immersion of DRCs. ^a,b,c^ Different lowercase superscript letters indicate differences in FS or FM before water immersion with the same composition and proportion (*p* < 0.05). ^A,B,C^ Different capital superscript letters indicate differences in FS or FM after water immersion with the same composition and proportion (*p* < 0.05). * Asterisk indicates a significant difference between before and after water immersion (*p* < 0.05). Bar graphs were created using GraphPad Prism 9.5.

The mean and standard deviation results for FS and FM of each DRC following different storage conditions are shown in Figure 6. Following each storage condition, the FBF appeared to be significantly stronger since it showed the greatest FS values in contrast to all BF-DRCs (*p* < 0.05). In addition, the FM of the FBF was considerably higher than all BF-DRCs following each storage condition (*p* < 0.05). For the experimental groups, the FS in each group did not change significantly with the increase in the filler content (*p* > 0.05). The FM increased with the increasing filler content (*p* < 0.05), and the FM of the UBT/Filler (30/70) and UBT/Filler (26/74) had no significant difference before and after water immersion (*p* > 0.05). All tested DRCs met the performance requirements of achieving 80 MPa FS, as stipulated by ISO 4049 [26].

**Figure 7:**
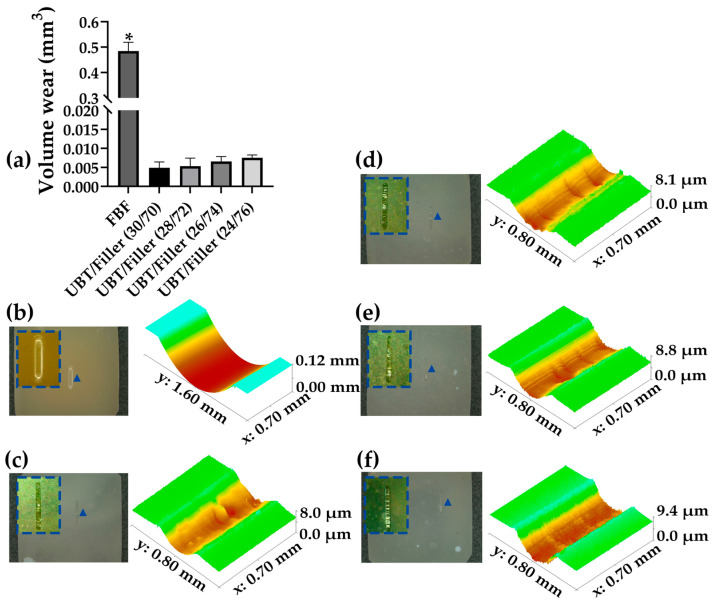
(**a**) Volume wear of dental resin composites (*p* = 0.000). (**b**–**f**) Surface morphology (triangle annotation) and 3D profile scanning images (20×) of samples after wear testing. The image within the dashed box shows an enlarged view of the wear marks at the triangular identifier. (**b**) DRCs of FBF, (**c**) DRCs of UBT/Filler (30/70), (**d**) DRCs of UBT/Filler (28/72), (**e**) DRCs of UBT/Filler (26/74), and (**f**) DRCs of UBT/Filler (24/76). * Asterisk indicates a significant difference between different groups (*p* < 0.05). Bar graphs were created using GraphPad Prism 9.5.

The volume wear results for the five resin materials are illustrated in Figure 7a. FBF demonstrated significantly higher volume wear (*p* < 0.05), whereas the other groups showed no significant difference (*p* > 0.05). The depth and morphology of all DRCs, examined through optical profilometry following the wear resistance test, are displayed in Figure 7b–f. Under the same friction load and cycles, FBF exhibited deeper and wider indentations. In contrast, the experimental groups showed shallower and lighter indentations. Increasing the filler content from UBT/Filler (30/70) to UBT/Filler (24/76) resulted in a slight rise in volumetric abrasion, yet this change lacked statistical significance (*p* > 0.05).

**Figure 8:**
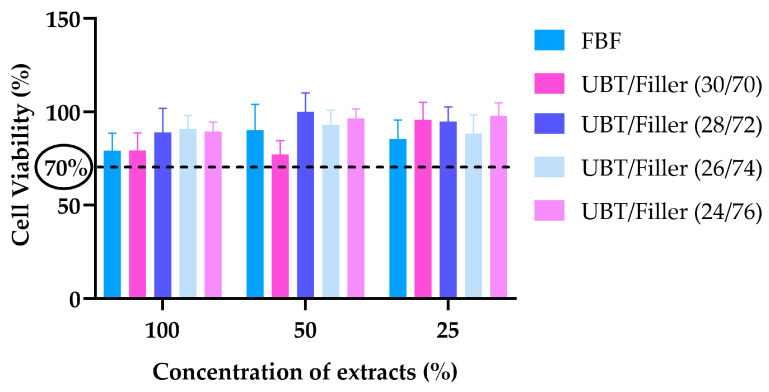
Cell viability of DRCs (100%, 50%, and 25% extract). The dashed line indicates that cell activity has reached 70%. Bar graphs were created using GraphPad Prism 9.5.

Figure 8 shows the results of the CCK-8 assay. The cell viability of all DRCs was greater than 70% at 100%, 50%, and 25% extract concentrations.

## 4. Discussion

DOC is a key parameter of DRCs, directly impacting their curing effectiveness. A notable feature of BF-DRC is its ability to deliver incremental fillings of 4 mm to 5 mm in a single treatment session, thereby reducing the clinical operation time [30]. Insufficient curing depth can cause more material to leach from poorly cured resin at the base of the filling, potentially irritating soft tissues and dental pulp, promoting bacterial growth, and triggering allergic reactions [31]. Enhancing the translucency of BF-DRCs is a crucial strategy for optimizing the curing process. The translucency of DRCs is influenced by the amount of filler and the refractive index variation between the filler particles and the resin matrix [32]. This disparity determines the extent of light penetration within the DRC. When the particle size of the filler is uniform, increasing the filler content generally leads to reduced translucency, a finding supported by most literature in this area [19,33]. Studies indicate that BF-DRCs with a lower filler content display significantly higher DOC than conventional DRCs, likely due to the minimal filler quantity aiding in polymerization and light transmission, thereby improving curing at greater depths [20]. In this study, the DOC of different materials was assessed using the scraping method described in ISO 4049. Under irradiation with a 550 mW/cm^2^ LED unit for 20 or 40 s, the tested DRCs met the standards for DOC in bulk-filled dental resin composites, all exceeding 4 mm. Additionally, the increased filler content did not significantly affect the DOC in the experimental group, leading to the rejection of the first null hypothesis.

Various alternative techniques, such as Vickers microhardness testing and infrared spectroscopy, can assess the DOC. Furthermore, it has been suggested that the ISO 4049 [26] scratch method may overstate the DOC [34]. Numerous studies have shown that the light transmittance of DRC shows a positive correlation with the VHN ratio of the bottom-to-top layers, suggesting a significant relationship between VHN and DOC [35,36]. In this study, the VHN bottom/top ratio of the 4 mm-thick DRC sample exceeded 80%, indicating that the tested sample meets the curing depth requirements for BF-DRCs [37]. Some DRCs exhibited bottom/top values greater than 100% at smaller thicknesses. This phenomenon may be attributed to the presence of an oxygen inhibition layer on the surface of the DRCs. Although a polyester film was employed during sample preparation to minimize atmospheric oxygen exposure, some oxygen may still affect the surface layer by generating radicals that can bond with resin monomers. These oxygen radicals can create stable peroxide radicals and are non-reactive, leading to incomplete polymerization of monomers on the resin surface, thus forming an oxygen-inhibition layer [38]. These factors subsequently impact the VHN at the surface of the DRCs. Moreover, this may relate to the post-curing of the samples, as flexible chains in the resin system can continue to polymerize at a certain rate for a short period after stopping the light exposure. The number of free radicals participating in post-curing at the sample bottom is significantly higher than at the top, making it difficult for the hardness ratio between the bottom and top to reflect the true curing effect. Reports indicate that the hardness of DRCs shows a significant improvement after 24 h of curing compared to 15 min, aligning with theoretical predictions of polymerization following irradiation within the resin [39].

VHN characterizes the resistance of DRCs to plastic deformation and wear. Research shows that the VHN of DRCs is influenced by the type, morphology, size, and content of filler particles. Increasing the filler content results in a higher VHN [40]. El-Safty et al. [41] investigated the nano-hardness of ten types of DRCs using nano-indentation, discovering a positive correlation between the nano-hardness of this series of resin composites and the filler loading. Jun et al. [42] studied 18 different DRCs to explore the relationship between the mechanical properties of various resin composite and filler fractions. They found that the surface hardness increased with the filler content, particularly when the filler particles were silanized, resulting in a significant increase in the hardness values of the DRCs with rising filler fractions. In this study, the increase in the content of inorganic fillers increased the VHN of the DRC, which further confirmed this view.

The polymerization process is intrinsically linked to the monomer chemistry, filler characteristics, photo-initiator concentration, and polymerization conditions [43]. Since the polymerization conditions, layer thickness, curing unit strength, and exposure time were standardized in this study, differences in the DC value of DRCs can be attributed to the different compositions of the materials, mostly to variations in the chemistry of their resin matrix and the filler loading. Research indicates that the DC decreased with an increase in the content of opaque fillers [44]. Higher DC values for DRC indicated lower levels of unreacted monomers within the resin composite and less cytotoxicity to the repair produced, with current resin composites having a final DC in the range of 50% to 80% [45]. The experiment revealed that the commercial control dental resin composite FBF possessed the lowest DC value. In contrast, the experimental groups (UBT/Filler system) demonstrated a higher DC, while no direct correlation was found between DC and filler content within the experimental groups. The variation in DC values might be more closely related to the chemical composition of the resin matrix, initial viscosity, and the flexibility of the monomer’s crystalline structure [46]. As the thickness of all DRC samples increased, a significant decline in DC was observed. This decrease occurred because the resin composite’s polymerization process was initiated by visible light, and the scattering of light by filler particles influenced the depth of light penetration. When thickness increments of 4–5 mm were applied, the effectiveness of the photo-initiator decreased in deeper regions due to light intensity attenuation, subsequently affecting the system’s polymerization and DC value. With an increase in inorganic filler content, the viscosity of the system rose, limiting the activity of high-molecular-weight free radicals, thereby restricting the chain growth step and leading to a decrease in DC value [47]. DC significantly impacts the mechanical properties and biocompatibility of DRCs. A high DC correlates with high polymerization shrinkage, while a low conversion rate relates to low mechanical performance, poor color stability, and reduced biocompatibility [45,48]. The testing of DC also provides data support for subsequent performance analysis.

Ideally, DRCs should exhibit high stability and waterproof characteristics. Water absorption emerges as a critical property influencing the clinical success of resin composite. It negatively impacts the hydrolytic stability of these materials, leading to discoloration [48], degradation of mechanical properties, reduced wear resistance, and hydrolytic degradation at the resin–filler interface [49]. WS and SL occur primarily when resin composites are exposed to aqueous environments, such as the oral cavity, allowing them to absorb water and release unreacted monomers. They are, therefore, related to the DC of the DRC; the higher the DC, the lower the number of unreacted monomers, leading to lower WS and SL values, while factors such as the type of monomer, filler content, and type also affect them [50]. The experimental data indicated that an increase in filler content contributed to a reduction in the water sorption (WS) of DRCs in the UBT/Filler system. Excluding the effects of DC, this reduction may relate to the water absorption capacity associated with the available equilibrium pore volume and the hydrophilicity of the resin monomers. As the inorganic filler content in resin composites increased, the concentration of hydrophilic resin monomers in the corresponding materials decreased, leading to an overall decline in WS. FBF exhibited higher WS and SL, likely due to its lower monomer conversion rate and the presence of smaller nanoparticle fillers, possessed a larger surface area, and could absorb more water. Likewise, smaller filler particles enhanced the filler/matrix interface area, increasing the resin composite’s susceptibility to water penetration and degradation [51].

VS during polymerization and the formation of interfacial gaps remain issues affecting the longevity of resin composite restorations. It is an unavoidable property of the polymerization process due to the short covalent bonds that result in a decrease in the distance between the monomer molecules, which reduces the total free volume within the monomer structure and produces a dense buildup of polymer molecules [52]. The size of the VS generates shrinkage stress, which affects the bonding interface between the tooth and the restoration. When the induced stress exceeds the adhesive strength between the bonding agent and the tooth, interfacial gaps will form [53]. Bis-EFMA-based BF-DRC achieves the purpose of reducing polymerization shrinkage by reducing the percentage of short double bonds through higher molecular weight. Experimental data indicated that the VS of DRCs in the UBT/Filler system decreased as the content of inorganic fillers increased. This trend may arise because the inorganic fillers maintain a constant volume before and after polymerization. Consequently, DRCs with a higher content of inorganic fillers exhibited lower VS under a given volume. SS arises from the shrinkage of DRCs under constrained conditions. This stress can lead to micro-gaps forming, resulting in micro-leakage of saliva and bacteria, degradation of the adhesive interface, secondary caries, pulpal changes, and ultimately clinical failure of restorations [54,55]. However, SS is not only related to the VS and DC of the DRCs but is also significantly associated with the elastic modulus during the formation of the polymer network [56,57]. Studies have evaluated the SS and elastic modulus of different BF-DRCs. They found that the SS of DRCs correlates with the composition of monomers and filler content. In addition, there is a notable correlation between SS and elastic modulus: a lower elastic modulus can facilitate stress dissipation during polymerization, while a higher elastic modulus can lead to higher shrinkage stress, even in low-shrinkage composites [58]. Figure 5 shows that the SS of UBT/Filler (24/76) and UBT/Filler (30/70) was high, likely linked to the system’s VS and FM. The high FM of UBT/Filler (24/76) hindered the dissipation of stress generated during polymerization [59], while the greater SS of UBT/Filler (30/70) was attributed to its higher VS. Reports indicate that SS positively correlates with the VS [60]. The differences between FBF and UBT/Filler systems stem from the distinct resin matrices and fillers used, which will not be discussed further in this paper. In conclusion, the increase in the inorganic powder filler content can significantly reduce the polymerization volume shrinkage of the system, so the third null hypothesis was rejected.

Extensive research has been conducted on FS and FM of DRCs to predict their performance in real clinical environments. Mechanical properties depend on factors such as the composition of the resin matrix, viscosity, and the type and content of fillers [61]. Studies indicate a correlation between the mechanical performance of DRCs and filler content [62,63]. Reports have assessed Bis-GMA/TEGDMA-based DRCs with filler loading levels ranging from 0 to 70 wt%. Research shows that the FM of DRCs increases exponentially with the rise in filler content. However, only minor differences in modulus were observed among DRCs containing 0–60 wt% fillers. In contrast, resins with 70 wt% fillers demonstrated significantly higher FM across all conversion levels [64]. It has also been reported that higher filler content does not always result in superior mechanical performance [65,66]. Fronza et al. characterized the effect of inorganic filler content in conventional DRCs (Herculite Classic, HER, 50 vol% filler content) and four types of BF-DRCs (Tetric EvoCeram Bulk Fill, TEC, 60 vol% filler content; Filtek Bulk Fill, FBF, 42.5 vol% filler content; EverX Posterior, EXP, 57 vol% filler content; Surefil SDR flow, SDR, 44 vol% filler content) on the biaxial flexural strength (FS) and flexural modulus (FM). The research found that although the FM of DRCs was influenced by filler mass fraction, the FS of EXP and TEC was lower than that of the lower-filler-loaded FBF and SDR [67]. The increase in polymer network density, stress transfer between filler particles and the resin matrix, and the adhesion between components also affected the degree of polymerization and the final performance of DRCs [68]. The experimental test results indicated that increasing the filler content did not have a significant impact on the FS of the UBT/Filler system. This was primarily because FS depends on the DC of DRCs rather than on the filler content or adequate silanization [69]. After immersion, the FS decreased slightly, but this difference was not statistically significant. Theoretically, prolonged soaking can impair the mechanical properties of the DRC. This deterioration occurs due to the degradation of the resin matrix, filler particles, or the silane layer at the interfaces. Water intrusion into the DRC may cause the hydrolytic breakdown of the silane layer, leading to debonding of filler particles and potentially their hydrolysis. The resin matrix swells and becomes plasticized upon water exposure. Thus, the flexural strength of the DRC will likely decline after soaking [50]. In this experiment, although the FS of the BF-DRCs decreased in numerical value after immersion, there was no statistically significant difference in FS before and after immersion. The increase in inorganic filler content significantly improved the FM of the DRC in the UBT/Filler system, a phenomenon supported by the theoretical analysis mentioned earlier [64]. The FM of UBT/Filler (28/72) significantly decreased before and after water immersion, possibly due to water molecules diffusing into the polymer network, functioning as a plasticizer, resulting in a decreased FM of DRCs. In contrast, the FM of UBT/Filler (24/76) increased significantly, while the higher filler contents in UBT/Filler (26/74) and FBF also showed an increasing trend in FM after immersion. This phenomenon may arise because the greater filler ratios in high- and medium-viscosity DRCs decrease the amount of resin vulnerable to water aging. Combined with the above, increasing the filler content could significantly improve the VHN and FM of Bis-EFMA-based BF-DRCs, so null hypothesis 2 was rejected.

Insufficient wear resistance of DRCs under chewing loads is a common reason for the premature failure of restorations. Generally, the variations in wear among the resin composite relate primarily to the volume and distribution of filler particles, the properties of the resin matrix, the degree of conversion, and the bonding between the resin matrix and filler [70]. It is theorized that DRCs with lower filler contents may exhibit higher wear values, as DRCs with higher filler contents have a greater surface Vickers hardness, which provides more protection to the resin matrix from filler abrasion, making the surface less susceptible to wear. However, Shimokawa et al. [71] revealed that DRCs with a high filler volume fraction showed more wear, while those with lower filler loadings experienced less. Hu et al. [72] investigated the fundamental wear behavior of resin composite with various filler contents under two-body wear conditions. Their study found that DRCs with a filler volume percentage below 60% had a lower two-body wear rate, while DRCs with a filler volume content of 80–87.5% exhibited higher wear rates. Under two-body wear conditions, incorporating highly loaded filler particles into the resin matrix decreased the wear resistance of DRCs. In this experiment, under the larger friction wear load of 25 N, the resin layer on the DRC surface, which is prone to damage, was rapidly worn away. This can lead to abrasion between the resin matrix and the exposed filler, with UBT/Filler (24/76), which has a higher filler content, experiencing more abrasion. In the current study, no significant relationship was found between filler content and wear amounts of DRCs, although the wear amounts across all experimental groups were notably lower than that of the commercial control FBF.

BF-DRCs are often employed for filling restorations in deeper cavities of posterior teeth due to their higher curing depth and other characteristics. Considering the unique features of the pulp–dentin complex, the filling materials must exhibit low cytotoxicity [73]. Ideally, DRCs should be non-toxic to both oral hard and soft tissues. However, numerous in vitro studies have indicated that the polymerization reaction of crosslinked polymer matrices generated by dimethacrylate resin monomers never completes 100%. The release of unpolymerized monomers can trigger cytotoxic reactions. In addition to the leaching of unreacted monomers, cytotoxicity may also stem from the release of initiators from the resin matrix or metal ions from inorganic fillers. This type of cytotoxic restorative material can elicit both short-term and long-term adverse tissue responses, ranging from postoperative sensitivity to irreversible pulp damage [73,74,75]. A study evaluated the cytotoxicity of flowable and paste-like BF-DRCs using real-time cell analysis, with conventional DRCs as a reference. The findings indicated that flowable and paste-like DRCs of the same brand exhibited similar cytotoxicity. Throughout the experiment, a significant reduction in cell viability was observed for BBF (Beautifil-Bulk Flowable) and BBR (Beautifil-Bulk Restorative). Considering that both DRCs contain pre-reacted glass-ionomer (PRG) fillers in their resin matrix, the higher fluoride release from PRG-filled DRCs compared to others suggests that the cytotoxicity of BBR and BBF may be linked to the fluoride and other metal ions released, such as aluminum, boron, sodium, silicon, strontium, and zinc, from the PRG fillers. This indicates that metal ions released from inorganic fillers in DRCs also significantly impact cytotoxicity [76]. Additionally, research has explored the correlation between filler content and the cytotoxicity of DRCs. Shajii et al. investigated the impact of inorganic filler content on the biodegradation of the Bis-GMA/TEGDMA resin system using cholesterol esterase. They found that the cumulative release of biodegradation products from DRC with 20 wt% filler was significantly higher than that from DRC with 40 wt% filler, indicating that the release of biodegradation products is related to the “filler/resin” ratio [77]. All the BF-DRCs involved in this study exhibited low cytotoxicity (with cell viability at 70% or higher), and no significant differences in cell viability were observed among the DRCs. Experimental data also indicated that the cell viability of FBF extracts was slightly lower than that of the UBT/Filler system. This difference may be attributed to the low solubility and high monomer conversion rate of the UBT/Filler system, resulting in a reduced release of unreacted monomers. The research demonstrated that an increase in inorganic filler content did not notably impact the cytotoxicity of BF-DRCs; however, considering that the variation in filler content within the system was minimal, the discussion on the effect of filler content on cytotoxicity remains limited. Overall, the UBT/Filler system exhibited lower cytotoxicity, indicating potential for clinical application as an oral restorative material.

## 5. Conclusions

Based on the results obtained in this study, the following conclusions were drawn:

1. The increase in inorganic filler content did not significantly affect the DOC, DC, SL, and cytotoxicity, and it could notably reduce the WS of the system of Bis-EFMA-based BF-DRCs.
2. The increase in inorganic filler content could significantly enhance the VHN and FM of Bis-EFMA-based BF-DRCs, aiding in the improvement of the mechanical properties of the system.
3. The increase in inorganic filler content could significantly reduce the VS of Bis-EFMA-based BF-DRC, to a certain extent, which could help improve the polymeric shrinkage of the system.
4. The UBT/Filler system with UBT/Filler (24/76) exhibited superior performance compared to commercial bulk-fill resin composite FBF in all aspects, except for flexural strength, flexural modulus, and polymerization shrinkage stress. While the flexural strength of UBT/Filler (24/76) was significantly lower than that of FBF, it still exceeded the minimum flexural strength requirement of 80 MPa, as stipulated by ISO 4049:2019(E), thereby meeting the mechanical performance criteria for posterior dental restorative materials.
5. Bis-EFMA-based BF-DRCs can achieve good operability, exceptional mechanical strength, and reduced polymerization shrinkage through an increase in filler content within a certain range. However, considering the prospects for clinical application, their biocompatibility requires further experimental discussion. Additionally, this study has limitations, such as testing the in vitro properties, which may not fully replicate the complexities of the oral environment. Moreover, the introduction of a single filler system cannot adequately balance the modulus of the system and polymerization shrinkage stress.

## Figures and Tables

**Table 1:** Components of resin systems for dental resin composites (DRCs).

DRC Experimental Groups	Components (wt%)					
	UDMA	Bis-EFMA	TEGDMA	CQ	DMAEMA	Fillers
UBT/Filler (30/70)	10.65	7.10	11.83	0.21	0.21	70
UBT/Filler (28/72)	9.94	6.62	11.04	0.20	0.20	72
UBT/Filler (26/74)	9.23	6.15	10.26	0.18	0.18	74
UBT/Filler (24/76)	8.52	5.68	9.46	0.17	0.17	76

**Table 2:** Depth of cure (DOC) of DRCs (curing time = 20 s and 40 s), as well as water sorption (WS) and solubility (SL) of DRCs (curing time = 40 s).

DRCs	DOC (mm) *n* = 6		WS and SL (μg/mm^3^) *n* = 6	
	DOC_20s_ (mm) (*p* = 0.451)	DOC_40s_ (mm) (*p* = 0.011)	WS (μg/mm^3^) (*p* = 0.000)	SL (μg/mm^3^) (*p* = 0.000)
FBF	4.197 ± 0.1 ^a^	5.018 ± 0.2 ^b^	33.27 ± 1.8 ^a^	13.65 ± 1.9 ^a^
UBT/Filler (30/70)	4.289 ± 0.1 ^a^	5.589 ± 0.2 ^a^	24.27 ± 1.3 ^b^	6.53 ± 0.7 ^b^
UBT/Filler (28/72)	4.290 ± 0.3 ^a^	5.427 ± 0.3 ^a,b^	25.74 ± 2.2 ^b^	6.81 ± 1.1 ^b^
UBT/Filler (26/74)	4.194 ± 0.2 ^a^	5.398 ± 0.3 ^a,b^	21.53 ± 0.2 ^c^	5.79 ± 1.1 ^b^
UBT/Filler (24/76)	4.415 ± 0.2 ^a^	5.660 ± 0.2 ^a^	21.66 ± 1.3 ^c^	5.76 ± 1.1 ^b^

^a,b,c^ Different lowercase superscript letters indicate differences within the same column (*p* < 0.05).

**Table 3:** Vickers microhardness (VHN) of DRCs (curing time = 40 s).

Height, mm	DRCs	VHN *n* = 9				
		Top (HV_0.5_)	*p*-Value	Bottom (HV_0.5_)	*p*-Value	Bottom/Top (%)
2	FBF	61.4 ± 0.2 ^a^	*p* = 0.000	58.4 ± 1.2 ^b^	*p* = 0.000	95.2
	UBT/Filler (30/70)	51.3 ± 1.3 ^c^		50.3 ± 0.7 ^d^		98.0
	UBT/Filler (28/72)	52.4 ± 0.5 ^c^		51.3 ± 0.9 ^d^		97.9
	UBT/Filler (26/74)	55.6 ± 1.2 ^b^		55.6 ± 1.2 ^c^		100.0
	UBT/Filler (24/76)	62.6 ± 1.4 ^a^		62.1 ± 1.7 ^a^		99.2
3	FBF	60.0 ± 1.2 ^b^	*p* = 0.000	58.9 ± 1.2 ^b^	*p* = 0.000	98.1
	UBT/Filler (30/70)	49.6 ± 0.3 ^e^		50.2 ± 1.3 ^c^		101.2
	UBT/Filler (28/72)	53.4 ± 2.0 ^d^		52.1 ± 1.5 ^c^		97.5
	UBT/Filler (26/74)	57.1 ± 0.5 ^c^		58.6 ± 1.5 ^b^		102.6
	UBT/Filler (24/76)	63.2 ± 1.6 ^a^		63.6 ± 0.1 ^a^		100.7
4	FBF	60.0 ± 1.2 ^b^	*p* = 0.000	55.7 ± 0.7 ^b^	*p* = 0.000	92.9
	UBT/Filler (30/70)	49.6 ± 0.6 ^e^		49.3 ± 0.9 ^d^		99.2
	UBT/Filler (28/72)	54.2 ± 1.1 ^d^		52.0 ± 1.5 ^c^		95.9
	UBT/Filler (26/74)	57.8 ± 1.6 ^c^		56.7 ± 0.6 ^b^		98.1
	UBT/Filler (24/76)	63.6 ± 0.1 ^a^		63.4 ± 0.8 ^a^		99.7
5	FBF	60.9 ± 1.7 ^b^	*p* = 0.000	51.0 ± 2.8 ^c^	*p* = 0.000	83.8
	UBT/Filler (30/70)	49.5 ± 0.7 ^d^		46.9 ± 0.9 ^b^		94.9
	UBT/Filler (28/72)	52.4 ± 0.7 ^c^		50.7 ± 0.7 ^c^		96.7
	UBT/Filler (26/74)	58.7 ± 2.8 ^b^		56.7 ± 0.5 ^b^		96.6
	UBT/Filler (24/76)	63.9 ± 1.0 ^a^		63.9 ± 1.0 ^a^		100.0

^a–e^ Different lowercase superscript letters indicate differences within the same column of the same thicknesses (*p* < 0.05).

**Table 4:** Double-bond conversion (DC) of DRCs (curing time = 40 s).

DRCs	DC (%)				
	2 mm (*p* = 0.000)	3 mm (*p* = 0.007)	4 mm (*p* = 0.000)	5 mm (*p* = 0.000)	*p*-Value
FBF	48.7 ± 1.7 ^b,A^	46.7 ± 1.9 ^b,A^	32.6 ± 7.7 ^b,A^	32.8 ± 2.1 ^b,A^	*p* = 0.092
UBT/Filler (30/70)	53.4 ± 1.9 ^a,A^	53.5 ± 3.0 ^a,A^	52.3 ± 4.4 ^a,A^	51.3 ± 2.5 ^a,A^	*p* = 0.743
UBT/Filler (28/72)	56.1 ± 1.0 ^a,A^	52.9 ± 2.6 ^a,A,B^	53.5 ± 3.5 ^a,A,B^	50.1 ± 3.5 ^a,B^	*p* = 0.042
UBT/Filler (26/74)	55.2 ± 1.5 ^a,A^	55.9 ± 2.6 ^a,A^	53.0 ± 3.0 ^a,A,B^	50.1 ± 2.4 ^a,B^	*p* = 0.013
UBT/Filler (24/76)	55.3 ± 1.0 ^a,A^	52.8 ± 2.7 ^a,A,B^	49.6 ± 2.3 ^a,B^	48.5 ± 4.0 ^a,B^	*p* = 0.005

^a,b^ Different lowercase superscript letters indicate differences within the same column of the same thicknesses (*p* < 0.05). ^A,B^ Different capitalized superscript letters indicate differences between thicknesses of the same sample (*p* < 0.05).

**Table 5:** Shrinkage stress (SS) and volumetric shrinkage (VS) of DRCs.

DRCs	SS (MPa) (*p* = 0.002)	VS (%) (*p* = 0.009)
FBF	1.60 ± 0.1 ^b^	4.91 ± 0.3 ^a,b^
UBT/Filler (30/70)	2.05 ± 0.2 ^a^	5.08 ± 0.6 ^a^
UBT/Filler (28/72)	1.93 ± 0.2 ^a^	4.86 ± 0.5 ^a,b^
UBT/Filler (26/74)	2.05 ± 0.2 ^a^	4.80 ± 0.8 ^b^
UBT/Filler (24/76)	2.16 ± 0.3 ^a^	4.65 ± 0.6 ^b^

^a,b^ Different lowercase superscript letters indicate differences within the same column (*p* < 0.05).

## Data Availability

The original contributions presented in the study are included in the article, further inquiries can be directed to the corresponding author/s.
